# Comment on Plutecki et al. The Anatomy of the Thoracic Duct and Cisterna Chyli: A Meta-Analysis with Surgical Implications. *J. Clin. Med.* 2024, *13*, 4285

**DOI:** 10.3390/jcm13195663

**Published:** 2024-09-24

**Authors:** Lomani A. O’Hagan, Anthony R. J. Phillips, John A. Windsor, S. Ali Mirjalili

**Affiliations:** 1 Department of Anatomy and Medical Imaging, University of Auckland, Auckland 1023, New Zealand; 2 Surgical and Translational Research Centre, Faculty of Medical and Health Sciences, University of Auckland, Auckland 1023, New Zealand; a.phillips@auckland.ac.nz (A.R.J.P.);; 3 Applied Surgery and Metabolism Laboratory, School of Biological Science, Faculty of Sciences, University of Auckland, Auckland 1010, New Zealand

We read, with interest, Plutecki and colleagues’ systematic review of the anatomy of the thoracic duct and cisterna chyli, recently published in JCM [1]. These important lymphatic structures are difficult to study, and the review has reasonably attempted to meta-analyze the varied and often disparate descriptions in the literature to identify any predominant morphological patterns.

Our group has, in recent years, published several reviews on this topic [2,3], as well as cadaveric and radiological research [4,5]. While one of our radiological publications [5] was included in the present review, we note that our cadaveric publication in 2020 was omitted [4]. Few cadaveric studies on the thoracic duct have been published in the last decade; this work was primarily concerned with the morphology of the terminal lymphovenous valve, but we also reported on the different patterns of the thoracic duct’s termination. We dissected the thoracic ducts in 12 cadavers: 7 were terminated via a single lumen, whilst 5 were found to branch prior to their venous confluence (2 bifurcate, 2 trifurcate, and 1 quadfurcate) [4].

Given that the mode of thoracic duct termination was one of the main anatomical characteristics meta-analyzed in the present review, the authors or other interested readers may find these cadaveric findings relevant.
